# Flagellated bacterial porter for *in situ* tumor vaccine

**DOI:** 10.15698/mic2022.09.784

**Published:** 2022-08-24

**Authors:** Haiheng Xu, Yiqiao Hu, Jinhui Wu

**Affiliations:** 1 State Key Laboratory of Pharmaceutical Biotechnology, Medical School of Nanjing University & School of Life Sciences, Nanjing University, Nanjing 210093, China.; 2 Jiangsu Provincial Key Laboratory for Nano Technology, Nanjing University, Nanjing 210093, China.; 3 Chemistry and Biomedicine Innovation Center, Nanjing University, Nanjing 210023, China.

**Keywords:** flagellate bacteria, antigen presentation, transport antigen, tumor vaccine, tumor periphery, dendritic cell, abscopal effect

## Abstract

Cancer immunotherapy, which use the own immune system to attack tumors, are increasingly popular treatments. But, due to the tumor immunosuppressive microenvironment, the antigen presentation in the tumor is limited. Recently, a growing number of people use bacteria to stimulate the body's immunity for tumor treatment due to bacteria themselves have a variety of elements that activate Toll-like receptors. Here, we discuss the use of motility of flagellate bacteria to transport antigens to the tumor periphery to activate peritumoral dendritic cells to enhance the effect of *in situ* tumor vaccines.

Cancer immunotherapy aims to harness the body's own immune system to fight cancer, generate a systemic immune response, restart and maintain the tumor-immune cycle, and restore the normal anti-tumor immune response [1]. As an effective cancer immunotherapy, *in situ* tumor vaccines have made great progress due to the identification of more and more tumor neoantigens [2, 3]. Radiotherapy and chemotherapy can promote the release of tumor antigens and increase the uptake and presentation of antigens by antigen-presenting cells (APC). However, due to the existence of immunosuppressive factors and immature dendritic cells (DCs) in tumors, the efficiency of antigen presentation is insufficient [4, 5]. Current methods to increase antigen presentation are mainly to increase the number and function of DCs, such as intratumoral injection of granulocyte macrophage colony stimulating factor (GM-CSF), Fms-like tyrosine kinase 3 ligand (Flt3L) or toll-like receptor agonists (such as CpG or poly I:C) [6]. But the immunosuppressive microenvironment has always existed, and newly recruited DC function remains repressed [5, 7, 8].

Published in Nature biomedical engineering, Jinhui Wu, Yiqiao Hu and co-authors demonstrated a new method of antigen presentation. Using the motility of bacteria, the antigens inside the tumor are captured and transported to the tumor periphery, and the normal functioning DCs in tumor periphery are activated, thereby generating a systemic anti-tumor immune response (**Fig. 1**) [9].

**Figure 1:**
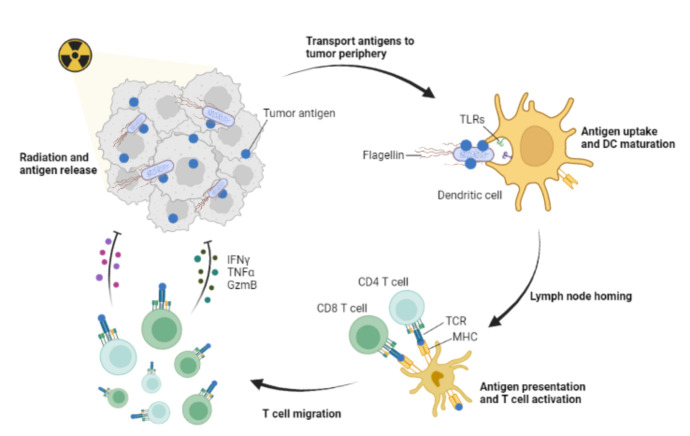
FIGURE 1: Systemic immune responses via the transport antigens to tumor periphery by Antigen-capture bacteria. The tumor antigen is released by radiotherapy. AC-Bacteria captures the released tumor antigen through electrostatic interaction, and then transports the tumor antigen to the tumor periphery through its own motion characteristics. The antigen is taken up by the functional DCs. The DCs then homed to the lymph nodes to present antigens and activate CD4+ and CD8+ T cells. T cells migrate to the tumor site through the vasculature and attack the tumor by secreting cytokines, etc. IFNγ, interferon-γ; TNFα, tumor necrosis factor α; GzmB, granzyme B; MHC, major histocompatibility factors; TCR, T cell receptor; TLRs, toll-like receptors.

Therapy with live tumor-targeting bacteria provides a unique option to cancer immunotherapy. The effectiveness of tumor-targeting bacteria is not directly affected by the ‘genetic makeup' of a tumor. Bacteria initiate their direct antitumor effects from deep within the tumor, followed by innate and adaptive antitumor immune responses [10]. Wu and co-authors used attenuated *Salmonella* Typhimurium (VNP20009) to transport antigens. VNP20009 was chromosomal deleted the *msbB* and *purl* genes. The deletion of the *purl* gene makes the bacteria unable to produce purines by themselves, so they tend to colonize high purine regions. The *msbB* gene reduces lipopolysaccharide (LPS) content by preventing the addition of a terminal myristyl group to the lipid A domain, thereby reducing LPS-induced systemic toxicity [11]. This unique bacteria has been evaluated in a phase-I clinical trial to treat non-reactive metastatic melanoma or renal cell carcinoma [11]. As a facultative anaerobic bacterium, VNP20009 can be significantly enriched in the tumor area after intravenous injection [12]. In order to increase the antigen capture properties of VNP20009, the authors engineered positively charged polyamide-amine dendritic polymer (PAMAM) onto the bacterial surface by electrostatic adsorption to obtain Antigen Capture-Bacteria (AC-Bacteria), which also uses electrostatic adsorption to adsorb tumor antigens released due to radiotherapy. The authors performed agar penetration and 3D cell spheroid penetration experiments with AC-Bacteria to demonstrate that nanoparticle-coated VNP20009 was able to capture antigens and still be motile.

Specific antigen presentation is core to immunotherapy. The authors demonstrated that AC-Bacteria can transport OVA257-264-specific antigen fragments to penetrate the agar membrane, enabling DCs to present OVA257-264-specific MHC fragments, indicating that DCs were activated by AC-Bacteria by capturing and transporting antigens, rather than DCs activation caused by the bacteria itself. Experiments were performed using dead bacteria without motility as controls. The authors combined fluorescently labeled AC-Bacteria with fluorescently labeled OVA antigen, and *in vivo* experiments proved that AC-Bacteria could indeed transport OVA antigen to a large number of CD103+ migrating DCs existing around the tumor periphery, and then CD103+ DCs were transported to lymph nodes and activated large numbers of DCs in lymph nodes. This implies that AC-Bacteria can efficiently capture specific antigens and activate antigen-specific DCs.

Tumor antigens released are the key to arousing immunity [13]. The authors detected the types and quantities of tumor antigens captured by AC-Bacteria by mass spectrometry. *In vivo*, the capture of antigens must compete with the very high concentration of serum albumin, fibrinogen, and IgG, among other proteins, *i.e.* non-tumor proteins. The authors performed radiotherapy on *in vivo* tumors in mice, and then prepared whole tissue lysate of tumor tissue for antigen capturing of AC-Bacteria. The results showed that compared with the control group (mPEG modification to shield the charge and no PAMAM modification), the number and types of antigens captured by the positively charged modified VNP20009 were significantly higher than other groups. And it can capture more tumor neoantigens and damage-associated molecular patterns (DAMPs). Overall, the AC-Bacteria designed by Wu and co-authors can capture the specific neoantigens inside the tumor, avoid the individual differences of tumor neoantigens caused by tumor heterogeneity, and expand the application scope of *in situ* vaccines.

The authors demonstrated that AC-Bacteria has a significant tumor suppressive effect on primary and distant tumors in multiple tumor models, *i.e.* melanoma (B16OVA, B16F10), colon cancer (CT26), breast cancer (4T1). AC-Bacteria can significantly increase the proportion of CD4+ T cells and CD8+ T cells in the tumor. And the immune memory effect can be maintained for about 240 days. This shows that this therapy can withstand the recurrence of the tumor for a long time, avoiding repeated treatment. Compared with the widely used adjuvant CpG oligonucleotide, AC-Bacteria has better therapeutic effect. So, AC-Bacteria has more than an adjuvant effect. The addition of CD8+ T cell antibody abolished the anti-tumor immune response induced by AC-Bacteria, indicating that the immunotherapy effect was due to cellular immunity induced by T cells. Since bacterial motility is caused by flagella, and flagella is the only activator of toll-like receptor 5 (TLR5), the presence of bacterial flagella can also further activate DCs, resulting in an enhanced immune response [14].

The expression or dysfunction of immune checkpoints is one of the important reasons for the occurrence of many diseases. The immune checkpoints of tumor cells are usually activated, so that antigens cannot be presented to T cells, and the normal immune function of T cells cannot be exerted, thereby implementing immune escape [15]. The authors combined PD-L1 and AC-Bacteria after radiotherapy, significantly inhibited tumor volume growth, and increased the ratio of CD4+ and CD8+ T cells to Regular T cells (Treg), indicating that the treatment of transferred antigens after radiotherapy can be synergistic with immune checkpoint therapy exert anti-tumor effect.

Wu and co-authors demonstrate that increasing peritumoral antigen presentation by transferring antigen from the intratumoral to the peritumoral region provides a new strategy for *in situ* tumor vaccines. Therefore, there are several key factors to pay attention to this strategy. First, the use of electrostatic binding to adsorb antigens may lead to nonspecific antigen capture and reduce the efficiency of antigen presentation. For the optimization of the capturing method of antigens, reducing the adsorption of non-antigen proteins *in vivo* is an important direction in the future. In addition, due to bacterial movement, the antigen presentation of peritumoral DCs increases. Modifying bacteria to enhance bacterial motility may further enhance the antigen presentation of DCs. So, the increase of bacterial flagella by gene editing may increase the motility of bacteria and further increase the presentation. Finally, the way of tumor antigen release can be extended from radiotherapy to other ways, such as chemotherapy, ultrasound therapy. This is also a key factor worthy of breakthrough.

All in all, Wu and co-authors show that low-toxic bacterial treatment can not only activate innate immunity, but also activate adaptive immunity and enhance the effect of tumor immunotherapy. Leveraging bacterial-specific motility to transport antigens to tumor periphery to activate DCs may open up new strategies for *in situ* vaccines.

## Abbriviations:

APC: – antigen-presenting cells;
DCs: – dentritic cells;
GM-CSF: – granulocyte macrophage colony stimulating factor;
Flt3L: – Fms-like tyrosine kinase 3 ligand;
LPS: – lipopolysaccharide;
PAMAM: – polyamide-amine dendritic polymer;
AC-Bacteria: – Antigen Capture-Bacteria;
DAMPs: – damage-associated molecular patterns;
TLR5: – toll-like receptor 5;
Treg: – Regular T cells.
